# The Influence of Divine Rewards and Punishments on Religious Prosociality

**DOI:** 10.3389/fpsyg.2016.01149

**Published:** 2016-08-03

**Authors:** James Saleam, Ahmed A. Moustafa

**Affiliations:** ^1^School of Social Sciences and Psychology, Western Sydney University, PenrithNSW, Australia; ^2^Marcs Institute for Brain and Behaviour, Department of Veterans Affairs, New Jersey Health Care System, Western Sydney University, PenrithNSW, Australia

**Keywords:** religion, psychology, priming, morality, prosociality, reward, punishment

## Abstract

A common finding across many cultures has been that religious people behave more prosocially than less (or non-) religious people. Numerous priming studies have demonstrated that the activation of religious concepts via implicit and explicit cues (e.g., ‘God,’ ‘salvation,’ among many others) increases prosociality in religious people. However, the factors underlying such findings are less clear. In this review we discuss hypotheses (e.g., the supernatural punishment hypothesis) that explain the religion-prosociality link, and also how recent findings in the empirical literature converge to suggest that the divine rewards (e.g., heaven) and punishments (e.g., hell) promised by various religious traditions may play a significant role. In addition, we further discuss inconsistencies in the religion-prosociality literature, as well as existing and future psychological studies which could improve our understanding of whether, and how, concepts of divine rewards and punishments may influence prosociality.

## Introduction

Among the most heated debates in moral philosophy is that between religious and non-religious people as to what constitutes ‘the good life’, and whether atheism or theism offers a sounder basis for ethics and morality (Craig, 2010; Law, 2011; Grayling, 2013). Theists often argue that godless worldviews provide no objective foundation for moral virtue (Craig, 2010), and that immorality and hedonism are likely consequences (Zacharias, 2004; Warren, 2014). However, many humanists have rebutted that the moral foundations of religion are, at least in part, rooted in self-interest. It is argued that theistic morality is confounded by the promise of divine rewards (e.g., eternal bliss) and punishments (e.g., eternal torment) as recompense for behaving in particular ways, and thus, that there is a hedonistic element inherent in religious morality (Stenger, 2011). This is an interesting objection, and there is a growing body of research that seems to support it, which we examine below.

## Prosociality and Religion

On the face of it, the claim of theologians and religious apologists seems to hold true. Numerous investigations have found that religious people behave more morally/prosocially^1^ than less (or non-) religious people. For example, in a large-scale study spanning over 70 countries, Stavrova and Siegers (2014) found that personal religiosity correlated positively with membership in charitable organizations. In fact, this effect was most pronounced in *non-theocratic* contexts; that is, contexts within which religious morals are not legally or socially enforced. Stavrova and Siegers (2014) also found that personal religiosity correlated negatively with the frequency of traffic offenses, the probability of purchasing stolen goods, and the probability of justifying the act of lying for a personal gain. It seems the more secular a society is, the greater the moral gap between more and less religious people is. Numerous other studies have also demonstrated links between personal religiosity and prosocial behavior (e.g., Regnerus et al., 1998; Saroglou et al., 2005; Heineck, 2014).

Priming studies contribute further evidence that religious people behave more prosocially than less (or non-) religious people. When reminded of God, or when primed with words relevant to God or religion, religious people have been found to behave more prosocially in anonymous economic games (Shariff and Norenzayan, 2007), to cheat less if they believe in punitive/vengeful deities (Shariff and Norenzayan, 2011), report greater ‘public self-awareness’ (Gervais and Norenzayan, 2012), and respond more socially desirably, that is, in ways which provide unrealistically positive information about oneself (Gervais and Norenzayan, 2012), among other such findings. In a recent meta-analysis, Shariff et al. (2016) found that while the effects of religious primes reliably increased the prosociality of people rated as being high in religiosity, such primes did not reliably affect less (or non-) religious people, suggesting that the effects of these primes were indeed due to their religious content.

While the religion-prosociality link makes sense, and many studies have found supporting evidence for it, the literature has not been one-sided. Galen (2012) provided a comprehensive critique of the state of research exploring the relationship between religious beliefs and prosociality. Galen noted that many studies exploring the religion-prosociality link have failed to find differences between religious and irreligious people (e.g., Bellemare and Kröger, 2007). Galen also noted that many studies have failed to distinguish between agnosticism, low/nominal religiosity, and irreligiosity; often combining these into a single group (e.g., ‘low religiosity’), which makes accurate comparisons of the prosocial attitudes/behaviors or religious and irreligious people difficult. Galen also showed that equivalent secular (e.g., ‘civil’) and religious (e.g., ‘divine’) primes identically influence prosociality (e.g., see Gervais and Norenzayan, 2012, Experiment 1; Shariff and Norenzayan, 2007; Harrell, 2012; Yilmaz and Bahçekapili, 2016), which suggests that religion per se is not the underlying cause of the increased prosociality; rather, religion exerts its effects indirectly, via its appeal to simpler cognitive and affective mechanisms (discussed below).

Despite his many concerns with the religion-prosociality literature, Galen (2012, p. 885) concluded that the evidence seems “fairly conclusive that priming religious concepts activates prosocial behaviors in participants”. However, the effectiveness of religious primes in promoting prosociality has also been questioned. While many studies have found a positive effect of religious priming on prosociality, others have failed to find a consistent effect. For example, Benjamin et al. (2010) failed to find an effect of religious priming on prosociality in the Dictator Game (in which individual players split a sum of money between themselves and a second player), and found that religious primes only led to increased contributions in a ‘public goods game’ (in which players are given a sum of money and can contribute some of that money to a public pot; the money in the pot is then multiplied and distributed equally across *all* participants, regardless of their personal contributions) from Protestants, but not Catholics, whose contributions actually decreased.

In their meta-analysis, Shariff et al. (2016) explored whether the religion-prosociality link was robust, after accounting for publication bias. While Shariff et al. (2016) found the link to be robust after controlling for publication bias, van Elk et al. (2016) obtained mixed results. Van Elk et al. (2016) tested the robustness of the religion-prosociality link using two distinct meta-analytic techniques: the Precision-Effect Testing–Precision-Effect-Estimate with Standard Error (PET-PEESE) technique (see Stanley, 2005; Stanley and Doucouliagos, 2007), and the Bayesian Bias Correction (BBC) technique (see Guan and Vandekerckhove, 2016). Only the latter demonstrated a robust effect of religion on prosociality. Accordingly, van Elk et al. (2016) concluded that meta-analytic techniques are useful, but have proven insufficient to settle the debate surrounding the religion-prosociality link. Hence, this is an area of continuing debate.

Another issue in the literature is that many religious priming studies have yielded antisocial effects (Galen, 2012). For example, Johnson et al. (2010) found that participants subliminally primed with Christian religious words displayed more covert racial prejudice and negative emotion toward African Americans than participants exposed to neutral primes. Another relevant finding in the literature (discussed in detail below) was that belief in a benevolent, forgiving and merciful God seemed to positively correlate with frequency of cheating on a quiz task (Shariff and Norenzayan, 2011). Using a variant form of the Dictator Game (involving allocation of stickers, rather than money), Decety et al. (2015) found that children from more religious families were significantly less generous than children from less religious or nonreligious families. Furthermore, despite the fact that children from religious families are expected to learn more about their religion as they age, older children from religious households in this study behaved *more* selfishly than younger children from religious households. Given these results, it may be more accurate to claim that religion seems to make people more prosocial in some instances, and more antisocial in others. Given the heterogeneity of specific beliefs across varying religious traditions, we should also expect to see variation in the effects of religious primes across religious traditions (Galen, 2012; also see Aveyard, 2014). If one religion claims that stealing from the wealthy is acceptable, and another dogmatically declares that theft is never acceptable, would a religious prime affect people from these two religions identically in a scenario which involved stealing from the wealthy to aid the poor?

While there is certainly a vast body of literature exploring *what* the differences may be between religious and less (or non-) religious people around the world in terms of prosocial attitudes and behaviors, such findings provide very limited insight into *why* such differences are often observed. If there is a link between religious belief and prosociality, then there must be an underlying reason for this. Stavrova and Siegers (2014) appealed to self-determination theory (see Deci and Ryan, 2000) to explain their findings, suggesting that the increased prosociality of religious people in more liberal contexts may be explained by the facts that (1) religions place a very heavy emphasis on morality and prosociality, and (2) “individuals are generally more committed to values (in this case, religious values) and tasks that they deem to be personally chosen rather than imposed by others” (Stavrova and Siegers, 2014, pp. 315-316). Religious texts certainly do promote prosociality (though they certainly also promote some antisocial attitudes and behaviors; e.g., Deuteronomy 20:10-14, 22:23-24; Ephesians 5:22-24; Qur’an 5:51, 9:5), but *why* do religious people adopt religious moral values in the first place? What is it about our religious traditions that makes them so morally persuasive?

## Predominant Hypotheses

Numerous hypotheses have been proposed to explain the apparent link between religious beliefs and prosociality. One such hypothesis is the ‘supernatural monitoring hypothesis’ (SMH), which posits that god-concepts may activate social systems in the human brain which could make believers feel as though they are being watched (Atkinson and Bourrat, 2011; also see Boyer, 2002). There are numerous studies demonstrating that being watched – or believing you are being watched – increases the likelihood of prosocial behaviors (e.g., Piazza et al., 2011; Nettle et al., 2013; Takagishi et al., 2015). If moral transgressions are observed, the observers may inform others, which could damage the reputation of the transgressor (Piazza and Bering, 2008; Shariff et al., 2014). Maintenance of one’s reputation provides a powerful incentive for prosocial behavior (Milinski et al., 2002; Piazza and Bering, 2008), and most of the world’s religions posit the existence of supernatural agents who constantly observe and judge our actions. According to the SMH, belief in such agents motivates religious people to behave prosocially (Boyer, 2002; Gervais and Norenzayan, 2012).

A related hypothesis is the ‘supernatural punishment hypothesis’ (SPH; see Johnson and Krüger, 2004; Johnson and Bering, 2006; Johnson, 2016). Proponents of the SPH argue that large-scale human cooperation (e.g., in large societies) is difficult to explain via appeal to concepts like kin altruism/selection, and direct/indirect reciprocity, and suggest that the emergence of beliefs in punitive supernatural agents, whether these arose as adaptations or exaptations, may better explain the data (Johnson, 2016; Yilmaz and Bahçekapili, 2016). If invisible, morally interested supernatural agents exist, they could be watching us at any time without our awareness. Indeed, the God of today’s main monotheisms apparently watches and judges *all* of us at *all* times (e.g., Job 34:21; Qur’an 49:18). If these agents are able to punish transgressors in this life or the next, it would be unwise to transgress, and on this basis, religious belief may promote prosociality (Johnson, 2005). Unlike the SMH (which pertains to reputation-management and social awareness), the SPH posits that the monitoring of supernatural agents will be particularly effective in promoting prosociality if those agents have the power to punish transgressors on earth (e.g., by making crops fail) or in the afterlife.

The SMH and SPH are not mutually exclusive, as reputation management and fear of punishment could concomitantly promote prosociality. Nor are these hypotheses the only possibilities. Harrell (2012) suggests that the reward-related aspects of religion can promote prosociality (this could be referred to as the ‘supernatural reward hypothesis’, or SRH). There are also hypotheses that are not centerd on the monitoring, generosity or punishments of supernatural agents. For example, some have argued that engagement in particular religious rituals acts as a *costly signal* which suggests to other adherents that the signaller can be trusted, as he/she belongs to the same group as them, and will endure great costs to demonstrate it (Irons, 2001; Sosis, 2004). For example, Jains will often spend considerable time carefully sweeping the paths in front of them to ensure they do not step/sit on any insects (Dundas, 2002), and Muslims will fast from sunrise until sunset during the month of Ramadan. Humans have an innate tendency toward coalitional thinking (Saad, 2011; Buss, 2014), and have been found to bond and cooperate on the basis of arbitrary and/or trivial similarities, often at the expense of outgroup members (e.g., Tajfel et al., 1971; also see Traulsen, 2008). This account may explain why religious prosociality has commonly been found only to extend to religious in-group members (see Galen, 2012).

Ultimately, prosocial behavior stems from multiple factors (Shariff et al., 2016), so it is unlikely that any single hypothesis will provide a comprehensive account of the religion-prosociality link. The empirical literature provides particular support for the SMH and SPH, and limited support (our literature search only located one such study: Harrell, 2012) for the SRH. This evidence will be explored below.

## Divine Rewards and Punishments

“*Honor your father and your mother, so that you may live long in the land the Lord your God is giving you*”.

– Exodus 20:12 [NIV]

“*For them who have done good is the best [reward] and extra. No darkness will cover their faces, nor humiliation. Those are companions of Paradise; they will abide therein eternally*”.

– Qur’an 10:26 [Sahih International Translation]

“*Moses said to the people, ‘Do not be afraid. God has come to test you, so that the fear of God will be with you to keep you from sinning*”.

– Exodus 20:20 [NIV]

“*Indeed, those who devour the property of orphans unjustly are only consuming into their bellies fire. And they will be burned in a Blaze*”.

– Qur’an 4:10 [Sahih International Translation]

There is a sound case to be made that if the observed differences between more religious and less (or non-) religious people are genuine, then they may derive – at least in part – from the fact that most religious traditions promise that moral behavior will be divinely rewarded, and immoral behaviors will be harshly punished (Johnson and Krüger, 2004; Harris, 2006; Baumard and Boyer, 2013; Johnson, 2016; Yilmaz and Bahçekapili, 2016). This is not to suggest that reward-anticipation and punishment-avoidance can completely account for why more and less (or non-) religious people differ in terms of prosociality (see above). The argument here is simply that reward-anticipation and fear of punishment form part of the overall picture, as to why religious people and less (or non-) religious people differ in terms of prosocial attitudes and behaviors, and recent findings in the literature (e.g., Harrell, 2012; Yilmaz and Bahçekapili, 2016; explored below) support this claim.

Given the preponderance of promises of divine rewards and punishments in today’s predominant monotheistic religions (i.e., all sects of Christianity, Judaism, and Islam), it is unsurprising that religion has commonly been linked to prosociality. Moral reciprocity – reward for moral behavior and punishment for immoral behavior – is a common theme in the vast majority of the world’s religions (Boyer, 2002; Johnson, 2005; Laurin et al., 2012; Hartberg et al., 2014). While believers may not recall specific scriptural verses regarding divine rewards and punishments for particular behaviors, the overarching message would be simpler to learn/internalize. The public statements of many popular religious apologists demonstrate the pervasiveness of this theme of moral reciprocity in religion. For example:

“*The Bible teaches that our time on earth is essentially preparation for eternity… This life is like a warm-up act, a dress rehearsal, for the real show in eternity. Once we fully grasp this, it makes all the difference in the world, affecting our choices, values, relationships, goals, and how we use our time and resources*”.

(Warren, 2009, p. 10)

Here is another example which conspicuously links religious morality to the anchor of eternity:

“*Virtually all conceptions of life after death, especially the religious conceptions, are rooted in the idea of cosmic justice… In all these doctrines, life after death is not a mere continuation of earthly existence, but rather a different kind of existence based on a settling of earthly accounts. These theories hold that even though we don’t always find terrestrial justice, there is ultimate justice. In this future accounting, what goes around does come around*”.

(D’Souza, 2009, p. 180).

It must be noted that religions vary in their precepts and injunctions greatly. Though not the focus of this paper, many Eastern religious systems (e.g., Buddhism and Jainism) appear to be much more selfless and benign than the predominant monotheisms of the West (though the vast majority of research in the field has focused on Christianity and Islam). While books like the Qur’an and the Old Testament are filled with references to divinely sanctioned violence, and the entitlement of certain chosen people to the belongings and labor of others, Buddhism and Jainism promote non-violence (e.g., Jains will even cover their mouths to lessen the chance that they will accidentally kill insects by swallowing them), open-mindedness, and self-discovery through meditation (Long, 2009; Harris, 2014). Despite this, concepts like karma and reincarnation still suggest a ‘give and get returned’ element of morality (D’Souza, 2009). Hence, it is likely that the promise of good karma and the threat of bad karma would promote moral behavior. Just as believing that immoral people are reincarnated as cockroaches would provide a powerful incentive for moral behavior. Indeed, Johnson (2016, p. 49) argued that the concept of karma may be particularly potent in promoting prosociality because karma is “embedded into the fabric of the universe”. That is, karma is a matter of absolute cause and effect, whereby moral actions lead to more positive outcomes, and negative actions lead to negative outcomes. Karma is not controlled by the whims of a forgiving and merciful deity; it is essentially a kind of supernaturalistic cause and effect.

The link between divine rewards/punishments and prosociality certainly makes theoretical sense, but now there is a growing body of empirical literature supporting the notion that such divine incentives do influence prosociality.

## Psychological Studies

There is a vast body of literature exploring the effects of implicit and explicit religious primes on prosociality (Shariff et al., 2016). However, one problem with the priming literature has been that the *different primes* being used, while all similar in that they are linked to religion (e.g., ‘heaven,’ ‘hell,’ ‘Jesus,’ ‘priest’), may also be different enough to produce *different effects* (Preston and Ritter, 2013). For example, ‘hell’ primes *may* elicit fear responses, while ‘heaven’ primes *may* improve mood or relieve anxiety. So while a pool of ‘religious primes’ may increase prosociality, it is difficult to isolate which specific primes (if not all) are underlying this effect.

The notion that related, but distinct, religious primes can have markedly different effects on prosocial behaviors and attitudes is supported by recent findings. Harrell (2012) found that reward-related secular (e.g., ‘applause,’ ‘admire’) or religious (e.g., ‘heaven,’ ‘salvation’) primes increased generosity in the Dictator Game significantly more than did neutral secular (e.g., ‘tree,’ ‘ocean’) or religious (e.g., ‘covenant,’ ‘temple’) primes. It seems plausible and likely that this effect occurred because the reward-related primes stimulated reward-anticipation in participants. However, one could also argue that this effect was due to the fact that the reward-related primes contained *positive* content, and that this altered the participants’ moods. Indeed, Pichon et al. (2007) found that religious primes with *positive* content (e.g., ‘bless,’ ‘praise’) increased prosocial intentions more than neutral religious words (e.g., ‘Bible,’ ‘parish’). However, many of the positive religious words in Pichon et al.’s (2007) study were relevant to divine rewards (e.g., ‘heaven,’ ‘salvation’), making their results difficult to interpret. Furthermore, all words used as primes by Harrell (2012) had been rated as being equally positive in a preliminary study; hence, it is more likely that the effects observed were due to reward-related content in primes, and were not due to positive content^2^.

Divine punishment primes have also been found to have a positive impact on prosociality. With a predominantly Muslim sample, Yilmaz and Bahçekapili (2016) found that participants primed with punishment-related religious and secular words (e.g., ‘devil’ and ‘prison’, respectively) reported more prosocial intentions than did participants primed with non-punishing religious and secular words (e.g., ‘spirit’ and ‘democracy’, respectively).^3^ In another study, Yilmaz and Bahçekapili (2016) found that participants who read sections of Qur’anic text related to divine punishment reported more prosocial intentions than did participants who read sections of Qur’anic text which highlighted Allah’s forgiveness and mercy. Hadnes and Schumacher (2012) also found evidence supporting the SPH in a sample from Burkina Faso. Participants who were primed with traditional beliefs and references to supernatural punishment (such punishments feature prominently in many traditional African belief systems; Hadnes and Schumacher, 2012) behaved more prosocially in the Trust Game (see Berg et al., 1995) relative to control participants. These results suggest that fear of divine punishment can motivate prosocial behavior.

This idea is supported by two studies conducted by Shariff and Norenzayan (2011), who found that participants’ views of God as either loving and caring, or angry and vengeful, were useful predictors of honesty in anonymous settings. Participants’ ratings of God as ‘forgiving,’ ‘kind,’ ‘gentle,’ and so on, correlated positively with the number of times they cheated during a mathematical quiz task (providing further evidence against the notion that positive aspects of religion promote prosociality); whereas participants’ ratings of God as ‘punishing,’ ‘vengeful,’ ‘terrifying,’ and so on, correlated negatively with the number of times they cheated. These effects were found regardless of whether participants rated the attributes of God before or after completing the quiz task, eliminating the possibility that ratings of God (i.e., as ‘kind’ vs. ‘vengeful’) were dependent on prior cheating during the quiz task.

Taken together, these findings suggest that reward-anticipation and fear of punishment play roles in motivating the prosocial attitudes and behaviors of religious people. The findings of Harrell (2012) and Yilmaz and Bahçekapili (2016) suggest that, even if only at the subconscious level, participants register the reward-relevance and punishment-relevance of certain religious words, and this influences their subsequent prosocial attitudes/behaviors. As was noted above, categorising primes is a difficult task, as there is likely to be considerable conceptual overlap (e.g., the word ‘salvation’ is positive *and* reward-related). Notably, Ritter and Preston (2013) demonstrated that people distinguish between words referring to religious agents, religious institutions (e.g., ‘church,’ ‘sermon,’ ‘baptism’), and more abstract religious concepts (e.g., ‘holy,’ ‘heaven,’ ‘faith’). Not enough words in the 32-word list provided to participants by Ritter and Preston pertained to reward or punishment, though ‘heaven’, ‘soul’, ‘miracle’, and ‘salvation’ appeared to cluster somewhat closely together, suggesting an underlying relation – arguably, their relevance to divine reward – between these terms.

Aside from the more direct investigations of the effects of divine-reward and divine punishment on prosocial behaviors, there are other findings worth citing which may add more indirect support for this notion. Kupor et al. (2015) found that priming people with notions of ‘God’ led to increased risk-taking when such risks were amoral (involved no moral/immoral content), but also that risk-taking behavior *decreased* when such risks involved *immorality*. Possibly there is an element of reciprocity here, whereby religious people expect God to give them good fortunes, in return for moral behavior (for a more expansive account of the idea of reciprocity in relationships with supernatural beings, see Boyer, 2002). Given that most religions warn of punishments for immoral behavior, it is unsurprising that participants were less willing to take immoral risks, and this makes sense in light of the SPH.

These data converge to lend support to the SPH and also to the notion that divine rewards might also be influential in the promotion of prosociality (the SRH). However, more work should be done to further investigate these hypotheses, using both behavioral and neuroimaging methods.

## Future Research

Although there are numerous studies (a) on links between prosocial behaviors and religion and (b) how divine rewards and punishments may partly account for why religious people behave more prosocially than less (or non-) religious people, to our knowledge, there is a lack of studies empirically linking these two streams. Furthermore, interpreting findings from some priming studies can be difficult (e.g., note the distinct conclusions of Pichon et al. (2007) and Harrell (2012); also see Preston and Ritter (2013). Here, we suggest potential psychological experiments, spanning vastly different methodologies, to probe the relationships between prosociality and divine rewards and punishments.

Firstly, as was suggested by van Elk et al. (2016), there is a dire need for largescale replication studies exploring the religion-prosociality link via the use of priming techniques. To reduce publication bias, van Elk et al. (2016) recommended that future replication studies also be pre-registered; that is, accepted for publication prior to the actual results of the study being obtained, as this would help to address the ‘file drawer problem’ (Rosenthal, 1979). The emphasis should always be on *testing* hypotheses, not on *confirming* them. While numerous priming studies have found links between religious devotion and attitudinal/behavioral prosociality (e.g., Shariff and Norenzayan, 2007), others have failed to find a consistent effect (e.g., Benjamin et al., 2010). Furthermore, the studies by Harrell (2012) and Yilmaz and Bahçekapili (2016) featured sample sizes of less than 160 people, and so these findings are in particular need of largescale replication.

Future work exploring the religion-prosociality link must also distinguish between irreligiosity and ‘low religiosity’ (see Galen, 2012), as this has also been an area of conceptual confusion, making the interpretation of the results of many previous studies a difficult task. Future work should also consider utilizing more diverse samples, exploring the religion-prosociality link in Buddhists, Hindus, among others, rather than relying overwhelmingly on Christian and Muslim samples as being representative of ‘religion’ generally. Religions vary considerably in their moral precepts and injunctions, and this should translate into differences in prosocial attitudes and behaviors. This can only be tested through studies utilizing sufficiently large samples, and sufficiently diverse samples, to allow for viable intergroup comparisons.

Future researchers should compare the effects of divine reward and punishment primes to discern which form of divine recompense is more influential when it comes to prosocial behavior. Many in the field have predicted that punishment-related religious primes would be more effective in promoting prosociality, as punishments are generally more effective than rewards in promoting prosociality (Johnson, 2005; Johnson and Bering, 2006; Shariff, 2008). Indeed, research has found that humans have what has been called a ‘negativity bias’; that is, humans are generally more attentive to negative stimuli than to positive stimuli (Baumeister et al., 2001; Johnson, 2016). However, this has not been empirically tested with regard to religious primes. Researchers could also examine whether divine reward and punishment primes promote prosociality through distinct avenues. This could be achieved, by priming one group of participants with both ‘heaven’ and ‘hell’ concepts, and comparing the effects on prosociality with those obtained from groups primed with divine reward *or* punishment concepts. If priming participants with both kinds of concept produces a greater effect on prosociality, then this would suggest that rewards and punishments influence prosociality via distinct mechanisms.

Future work should also investigate whether divine punishment primes cause fear. This can be accomplished by measuring Galvanic Skin Response (GSR), which is defined as a change in the electrical conductivity of the skin (i.e., with the onset of the prime), and has been used extensively to measure fear in psychological experiments (Williams et al., 2001). Researchers could compare the effects of subliminal/implicit primes relevant to supernatural punishments with neutral religious primes, and primes relevant to supernatural agents (e.g., ‘God,’ ‘Jesus,’ ‘Muhammad’) to see whether particular types of primes produce different physiological effects. This would provide further evidence for the notion that distinct kinds of religious primes have distinct effects (Preston and Ritter, 2013). Researchers could also test whether GSR in the agent-prime condition is mediated by participants’ views of the relevant religious agents (e.g., vengeful or merciful).

Finally, future research should utilize neuroimaging techniques to increase our understanding of how/where primes are having their effects. By using neuroimaging techniques, we can examine patterns of neural activation during the priming process. If reward-related religious primes truly are promoting prosociality via the avenue of reward-anticipation, then one would predict that participants exposed to such primes would show increased activation in the ventral striatum and orbitofrontal cortex, brain areas that have been repeatedly shown to be activated in response to reward (Olds and Milner, 1954; Young et al., 1998; Knutson and Cooper, 2005; Abe and Greene, 2014). If punishment-related religious primes are promoting prosociality by making people fear divine punishment, one would predict increased activity in the amygdala, a brain region that has repeatedly been shown to activate in response to punishment (LeDoux, 1993; Orsini and Maren, 2012; Moustafa et al., 2013). If the expected findings are obtained, this would provide neural evidence in support of current interpretations of the priming data. However, if the expected findings were not obtained, then perhaps we would need to re-evaluate how religious primes are being categorized (e.g., ‘reward-related’ vs. ‘positive’). Or perhaps null findings may reflect more on the shortcomings of particular priming paradigms.

Researchers considering a neuroimaging approach to exploring the effects of religious primes could explore the effects of subliminal, implicit and/or explicit primes. While subliminal primes have been found to be effective in eliciting subcortical neural activity, subliminal lexical primes (e.g., words) have tended to produce small effects (Brooks et al., 2012). It is also worth noting that implicit priming has not always proven effective in producing effects detectable by fMRI. For example, Powers and Heatherton (2013) failed to find a priming effect at the neural level using fMRI, despite using an implicit priming paradigm (the ‘sentence unscrambling task’) which has often been found to produce behavioral effects (e.g., see Sommer and Baumeister, 2002; Fitzsimons and Bargh, 2003; Shariff and Norenzayan, 2007; DeWall and Bushman, 2009; though also see Doyen et al., 2012; Aveyard, 2014). Though null effects are still meaningful, such findings in fMRI studies do not constitute definitive proof that primes failed to produce effects. At best, we could only conclude that *if* there actually was an effect, it was not detectable using fMRI technology (Powers and Heatherton, 2013), which is still a valuable insight.

## Conclusion

The explosion of studies exploring possible links between religion and prosociality has given rise to a plethora of interesting hypotheses and controversies. Some means by which these controversies could possibly be addressed have been outlined (e.g., utilizing GSR to test for physiological effects of particular religious primes). If religion truly does promote prosociality, then there must be reasons for this. The SMH, SPH and SRH make theoretical sense, and recent priming studies offer early support for these hypotheses. However, there is much work that needs to be done. These confirmatory findings should be accepted tentatively until largescale – preferably pre-registered – replications have been conducted. If these findings are replicated, future researchers should conduct experiments (e.g., using GSR or fMRI) aimed at uncovering how particular primes are affecting participants. These insights may contribute to an overall picture of how and why reward-related and punishment-related religious primes influence prosocial behavior, if indeed they do.

## Author Contributions

All authors listed, have made substantial, direct and intellectual contribution to the work, and approved it for publication.

## Conflict of Interest Statement

The authors declare that the research was conducted in the absence of any commercial or financial relationships that could be construed as a potential conflict of interest.

## Footnotes

1 The terms ‘morality’ and ‘prosociality’ are often used interchangeably in the literature. Prosociality has largely been broken down into two distinct constructs: prosocial attitudes and prosocial behaviors. Prosocial attitudes are those which reflect a desire and willingness to help others. Prosocial behaviors are those which benefit/help others (regardless of the motivation; hence, such behaviors are not *necessarily* ‘moral’). For clarity and consistency, this paper tends to refer to prosociality, instead of morality.

2 It is also worth noting that Yilmaz and Bahçekapili (2016; reviewed below) used a list of largely *positive* religious words (e.g., ‘grace’, ‘mercy’, ‘heaven’) in their pool of *non-punishing* religious primes. Non-punishing primes did not significantly increase prosocial intentions. In their second study, reading collections of Qur’anic verses which emphasized the *positive* and non-punitive qualities of Allah did not increase prosociality. These findings contrast with the findings of Pichon et al. (2007).

3 It should be noted that, in study 1, Yilmaz and Bahçekapili (2016) did not utilise Bonferroni corrections to their alpha-criterion for multiple comparisons, and so there is an increased chance of a type 1 error. This should be considered when interpreting their results.
